# Supplementary material for: Ordered arrangement of dendrites within a C. elegans sensory nerve bundle
Source: eLife. 2018 Aug 20;7:e35825. doi: 10.7554/eLife.35825 (PMC6133548; doi:10.7554/eLife.35825)
Supplement: Supplementary file 1. [file elife-35825-supp1.docx]

## Supp. File 1. Strains used in this study

| **Strain** | **Genotype** | **Figure first referenced** |
| --- | --- | --- |
| CHB1963 | *hmnIs23 III; hmnIs17 V* | Fig. 1 |
| CHB2429 | *hmnIs23 III; hmnEx1328* | Fig. S1 |
| CHB2305 | *daf-19(m86)II; hmnIs23 III; hmnIs17 V; (sa204)X* | Fig. 3 |
| CHB2044 | *mec-8(u74)I; hmnIs23 III; hmnIs17 V* | Fig. 3 |
| CHB2258 | *hmnIs23 III; hmnIs17 V; hmnEx1234* | Fig. 3 |
| CHB284 | *dig-1(n1321)III* | Table 1 |
| CHB1000 | *ptp-3(mu256)II* | Table 1 |
| CHB286 | *sax-7(ky146)IV; kyIs4 X* | Table 1 |
| CHB182 | *vab-1(dx31)II* | Table 1 |
| CHB178 | *sax-3(ky123)X* | Table 1 |
| CHB597 | *cdh-4(rh310) rhIs4 III* | Table 1 |
| CHB2245 | *trIs78; nrx-1(wy778)V* | Table 1 |
| CHB169 | *unc-40(e271)I* | Table 1 |
| CHB994 | *dma-1(wy686)I; wyIs378 X* | Table 1 |
| CHB1006 | *ptp-3(ok244)II* | Table 1 |
| CHB848 | *igcm-1(ok711)X* | Table 1 |
| CHB1005 | *plx-2(ev773)II; him-5(e1490)V* | Table 1 |
| CHB1020 | *nlr-1(tm2050)IV/nT1[qIs51] IV; +/nT1 V* | Table 1 |
| CHB2244 | *wyIs592 III; mnr-1(wy758)V* | Table 1 |
| CHB1202 | *ptp-4(gk715362)IV; hmnEx598* | Table 1 |
| CHB999 | *kyIs235 V; syg-1(ky652)X* | Table 1 |
| CHB596 | *rhIs4 hdIs26 III; fmi-1(rh308)V* | Table 1 |
| CHB1008 | *casy-1(ok739)II* | Table 1 |
| CHB1007 | *scd-2(ok565)V* | Table 1 |
| CHB1010 | *rig-3(ok2156)X* | Table 1 |
| CHB998 | *dgn-1(cg121)X; cgEx308* | Table 1 |
| CHB2647 | *cdh-4(rh310)III; hmnIs17 V; hmnEx1486* | Fig. 4 |
| CHB2646 | *hmnIs17 V; hmnEx1486* | Fig. 4 |
| CHB2087 | *ptp-3(mu256)II; hmnIs23 III; hmnIs17 V* | Fig. 5 |
| CHB1840 | *hmnEx1021* | Fig. 5 |
| CHB2060 | *hmnIs23 III; sax-7(ky146)IV; hmnIs17 V* | Fig. 5 |
| CHB1687 | *hmnEx912* | Fig. 5 |
| CHB2116 | *ptp-3(ok244)II; hmnIs23 III; hmnIs17 V* | Fig. 5 - Supp. Fig. 1 |
| CHB2526 | *hmnIs23 III; sax-7(eq1)IV; hmnIs17 V* | Fig. 5 - Supp. Fig. 1 |
| CHB3095 | *hmnIs23 III; sax-7(nj53) IV; hmnIs17 V* | Fig. 5 - Supp. Fig. 1 |
| CHB2350 | *ptp-3(mu256)II; hmnIs23 III; sax-7(ky146)IV; hmnIs17 V* | Fig. 5 - Supp. Fig. 1 |
| CHB2283 | *dma-1(wy686)I; hmnIs23 III; hmnIs17 V* | Fig. 5 - Supp. Fig. 2 |
| CHB2503 | *clr-1(e1745)II; hmnIs23 III; hmnIs17 V* | Fig. 5 - Supp. Fig. 2 |
| CHB2155 | *hmnIs23 III; hmnIs17 V; igcm-1(ok711)X* | Fig. 5 - Supp. Fig. 2 |
| CHB2525 | *hmnIs23 III; hmnIs17 V; hmnEx1416* | Fig. 6 |
| CHB3097 | *hmnIs23 III; hmnIs17 V; hmnEx1746* | Fig. 6 |
| CHB2407 | *hmnIs23 III; hmnIs17 V; hmnEx1291* | Fig. 6 |
