# Supplementary material for: Ordered arrangement of dendrites within a C. elegans sensory nerve bundle
Source: eLife. 2018 Aug 20;7:e35825. doi: 10.7554/eLife.35825 (PMC6133548; doi:10.7554/eLife.35825)
Supplement: Supplementary file 2. [file elife-35825-supp2.docx]

## Supp. File 2. Transgenes used in this study

**Integrated:**

| **Allele(s)** | **Constructs** | **References and Notes** |
| --- | --- | --- |
| *hdIs26III* | *odr-2*pro:CFP, *sra-6*pro:DsRed2 | (Steimel et al., 2010) |
| *hmnIs17 V* | pCY31, pMH130, pMH91 | This study |
| *hmnIs23 III* | pCY30, pDPMM051 | This study |
| *kyIs4 X* | *ceh-23*pro:GFP, *lin-15(+)* | (Zallen et al., 1999) |
| *kyIs235 V* | *odr-1*pro:RFP, *unc-86*pro:VAMP-YFP, *unc-4*pro:*lin-10*-RFP intron | (Shen and Bargmann, 2003) |
| *rhIs4 III* | *glr-1*pro:GFP, *dpy-20(+)* | (Schmitz et al., 2008; Steimel et al., 2010) |
| *trIs78* | pPRGS698, pPRGS699, pPRGS382 | (Maro et al., 2015) |
| *wyIs378 III* | pOL020, pOL090, *odr-1*pro:RFP | (Liu and Shen, 2011) |
| *wyIs592 III* | pOL020, *odr-1*pro:RFP | (Dong et al., 2013) |

**Extrachromosomal:**

| **Allele(s)** | **Constructs** | **References and Notes** |
| --- | --- | --- |
| *cgEx308* | pJK600, pJK602, pRF4 | (Johnson et al., 2006) |
| *hmnEx598* | pCY13, pCY14, pMH91, pMH130 | This study |
| *hmnEx912* | pCY74, pCY134 | This study |
| *hmnEx1021* | pCY74, pCY152 | This study |
| *hmnEx1234* | pBS, pCY167, pCY168, pIL53 | This study |
| *hmnEx1291* | pBS, pCY168, pCY190 | This study |
| *hmnEx1328* | pCY50, pCY56, pCY168 | This study |
| *hmnEx1416* | pCY118, pCY168 | This study |
| *hmnEx1486* | pCY30, pCY168 | This study |
| *hmnEx1746* | pCY191, pCY168 | This study |
