# Supplementary material for: Ordered arrangement of dendrites within a C. elegans sensory nerve bundle
Source: eLife. 2018 Aug 20;7:e35825. doi: 10.7554/eLife.35825 (PMC6133548; doi:10.7554/eLife.35825)
Supplement: Supplementary file 3. [file elife-35825-supp3.docx]

## Supp. File 3. Plasmids used in this study

| **Plasmid** | **Description** | **Notes** |
| --- | --- | --- |
| pBS | Bluescript |  |
| pCY13 | *odr-10*pro:CFP | This study |
| pCY14 | *gcy-8*pro:YFP | This study |
| pCY30 | *odr-10*pro:YFP | This study |
| pCY31 | *gcy-8*pro:CFP | This study |
| pCY50 | *odr-1*pro:CFP | This study |
| pCY56 | *ops-1*pro:mCherry | This study |
| pCY74 | *pha-4*pro:YFP | This study |
| pCY118 | *odr-10*pro:SAX-7S | This study; SAX-7S cDNA was a gift of Hiroyuki Sasakura and Ikue Mori (Sasakura et al., 2005) |
| pCY134 | *sax-7*pro:NLS-mCherry-NLS | This study |
| pCY152 | *ptp-3b*pro:NLS-mCherry-NLS | This study |
| pCY167 | *F16F9.3*pro:DTX | This study |
| pCY168 | *unc-122*pro:RFP | This study |
| pCY190 | *osm-6*pro:SAX-7S | This study |
| pCY191 | *ptp-3b*pro:SAX-7S | This study |
| pDPMM051 | *unc-119*(+) | (Maduro and Pilgrim, 1995) |
| pIL53 | *F16F9.3*pro:mApple | I. Low, unpublished |
| pJK600 | *dgn-1* genomic region | (Johnson et al., 2006) |
| pJK602 | *dgn-1*pro:GFP | (Johnson et al., 2006) |
| pMH91 | *gcy-7*pro:mCherry | This study |
| pMH130 | *gcy-5*pro:mCherry | This study |
| pOL020 | *ser-2*prom3:myrGFP | (Dong et al., 2013) |
| pOL090 | *rab-3*pro:mCherry | (Liu and Shen, 2011) |
| pPRGS382 | *myo-2*pro:mCherry | (Maro et al., 2015) |
| pPRGS698 | *unc-129*msp:MADD-4A::YFP | (Maro et al., 2015) |
| pPRGS699 | *unc-129*msp:MADD-4B::YFP | (Maro et al., 2015) |
| pRF4 | *rol-6*(*su1006*) | (Mello et al., 1991) |
